# Placotylene A, an Inhibitor of the Receptor Activator of Nuclear Factor-κB Ligand-Induced Osteoclast Differentiation, from a Korean Sponge *Placospongia* sp.

**DOI:** 10.3390/md12042054

**Published:** 2014-04-03

**Authors:** Hiyoung Kim, Kwang-Jin Kim, Jeong-Tae Yeon, Seong Hwan Kim, Dong Hwan Won, Hyukjae Choi, Sang-Jip Nam, Young-Jin Son, Heonjoong Kang

**Affiliations:** 1 Center for Marine Natural Products and Drug Discovery, School of Earth and Environmental Sciences, Seoul National University, NS-80, Seoul 151-747, Korea; E-Mails: reihyoung@snu.ac.kr (H.K.); dongfal2@snu.ac.kr (D.H.W.); 2 Department of Pharmacy, Sunchon National University, 315 Maegok-dong, Suncheon, Jeollanam-do 540-742, Korea; E-Mail: mastiffk@naver.com; 3 Research Institute of Basic Science, Sunchon National University, Suncheon 540-742, Korea; E-Mail: sa-dou@hanmail.net; 4 Laboratory of Translational Therapeutics, Pharmacology Research Center, Division of Drug Discovery Research, Korea Research Institute of Chemical Technology, Daejeon 305-600, Korea; E-Mail: hwan@krict.re.kr; 5 College of Pharmacy, Yeungnam University, 214-1 Dae-dong, Gyeongsan 712-749, Korea; E-Mail: h5choi@yu.ac.kr; 6 Department of Chemistry and Nano Science, Global Top 5 Program, Ewha Womans University, Seoul 120-750, Korea; E-Mail: sjnam@ewha.ac.kr; 7 Research Institute of Oceanography, Seoul National University, NS-80, Seoul 151-747, Korea

**Keywords:** *Placospongia*, sponge, RANKL, osteoclasts

## Abstract

A new inhibitor, placotylene A (**1**), of the receptor activator of nuclear factor-κB ligand (RANKL)-induced osteoclast differentiation, and a regioisomer of placotylene A, placotylene B (**2**), were isolated from a Korean marine sponge *Placospongia* sp. The chemical structures of placotylenes A and B were elucidated on the basis of 1D and 2D NMR, along with MS spectral analysis and revealed as an iodinated polyacetylene class of natural products. Placotylene A (**1**) displayed inhibitory activity against RANKL-induced osteoclast differentiation at 10 μM while placotylene B (**2**) did not show any significant activity up to 100 μM, respectively.

## 1. Introduction

Halogenated marine natural products have possessed structural diversity and displayed astonishing biological activities [1]. More than 2000 halogenated compounds have been isolated from marine organism as queried on Antimarin707 and Scifinder databases. In particular, polyacetylene marine natural products are commonly found to contain halogen atoms [2,3,4,5,6]. Only two classes of iodinated marine natural products have been reported as iodinated polyacetylenes. Phosphoiodyns A and B, the first iodinated polyacetylenes of marine natural products with a unique C-P bond, isolated from a marine sponge *Placospongia* sp. [7]. Four iodinated acetylenic acids were recently isolated from two marine sponges *Suberites* spp. [8]. These iodinated marine natural products have anti-obesity and anti-inflammatory effects, respectively. 

Bone homeostasis is maintained by the action of bone-resorbing osteoclasts and bone-forming osteoblasts. The balance between the activities of osteoclasts and osteoblasts is important in preserving bone mass. The balance can be broken by overactive osteoclasts that cause bone diseases, such as osteoporosis and multiple myeloma characterized by excessive bone loss [9]. 

Osteoclasts, which originate from bone marrow-derived macrophages (BMMs) of hematopoietic lineage, are mainly responsible for bone resorption, and play essential roles in maintaining skeletal homeostasis [10]. In the differentiation of osteoclasts, BMMs are mainly regulated by receptor activator of nuclear factor-κB ligand (RANKL) and macrophage colony-stimulating factor (M-CSF) for their differentiation and for their proliferation and survival, respectively [11,12]. 

The RANKL induces the nuclear factor of activated T cells c1 (NFATc1), which is a well-known master transcription factor for osteoclastogenesis [13]. Osteoclastogenesis involves complex processes, including differentiation to form tartrate-resistant acid phosphatase (TRAP)-positive cells, fusion to form multinucleated cells, and activation to resorb bone. Osteoclast differentiation-related factors are expressed during these processes, including TRAP, cathepsin K, and dendritic cell–specific transmembrane protein (DC-STAMP) [14,15,16].

**Figure 1:**
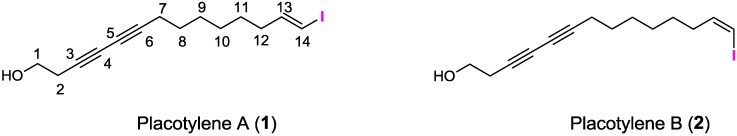
The chemical structures of placotylenes A and B.

As part of a collaborative program to identify inhibitors of osteoclast differentiation, screening of marine natural product library led to a new compound placotylene A (**1**) along with placotylene B (**2**) which is a derivative of placotylenes A, being isolated from a marine sponge, *Placospongia* sp. Details of the structure elucidation and biological activity of placotylenes A (**1**) and B (**2**) are described herein (Figure 1). 

## 2. Results and Discussion

### 2.1. Structure Elucidation

**Figure 2:**
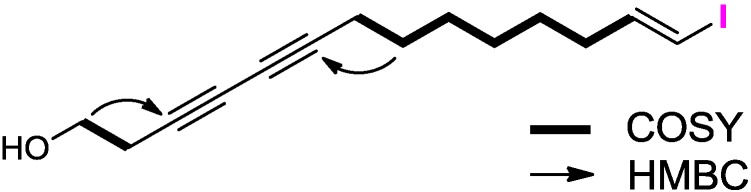
COSY (bold) and key HMBC (arrow) correlations of **1**.

The molecular formula of placotylene A (**1**), was deduced as C_14_H_20_OI based on the molecular ion peak at *m/z* 331.0561 [M + H]^+^ in the HRCIMS and ^13^C NMR data. Thus, the compound **1** had five degrees of unsaturation. The IR spectrum of **1** showed characteristic absorption bands at 3352 cm^−1^ indicating the presence of a hydroxy group. The ^1^H NMR spectrum of **1** displayed two methines (δ_H_ 6.50 and 5.98), eight methylenes (δ_H_ 1.38, 1.29, 1.39, 1.51, 2.05, 2.25, 2.53, and 3.75) including one oxygenated methylene (δ_H_ 3.75), and one hydroxy protons (δ_H_ 1.96). Interpretation of the ^13^C NMR and HSQC spectroscopic data indicated four characteristic carbon signals for acetylene groups (δ_C_ 65.1, 67.1, 73.8, and 78.1), olefinic carbons (δ_C_ 74.5 and 146.5), an oxygen-bearing carbon (δ_C_ 60.4), and seven methylene carbons (δ_C_ 19.0, 23.6, 28.1, 28.1, 28.3, 28.5, and 35.9). COSY crosspeaks [H-1/H-2 and H-7/H-8/H-9/H-10/H-11/H-12/H-13] allowed two distinct fragments, with two and seven carbon units, to be identified (Figure 2). These two fragments were assembled by HMBC correlations. Three bond HMBC correlations from H-1 to C-3, and from H-8 to C-6, allowed the C-2/C-3 and C-6/C-7 attachments, to be identified. Lastly, the [M − I]^+^ ion peak at *m/z* 203 in the MS data and the comparison of the chemical shifts of H-13 (δ_H_ 6.50 and δ_C_ 146.5) and H-14 (δ_H_ 5.98 and δ_C_ 74.5) to those of the known iodinated vinyl proton of (*E*)-1-iodo-1-octene supported the location of the iodine atom at the C-14 terminus [17]. Thus, the structure of placotylene A (**1**) was determined to be (*E*)-14-iodotetradeca-13-en-3,5-diyn-1-ol.

The molecular formula of placotylene B (**2**) was deduced to be C_14_H_20_IO, which is the same as the formula of placotylene A (**1**) based on the molecular ion peak at *m/z* 331.05621 [M + H]^+^ in the HRFABMS and ^13^C NMR data. The ^1^H and ^13^C data are almost identical to those of placotylene A, except for the coupling constants of the H-13 (δ_H_ 6.19, dt, *J* = 14.3, 7.3 Hz) and the H-14 (δ_H_ 6.27, br d, *J* = 7.3 Hz), indicating the (*Z*) geometry of C-14. Therefore, placotylene B (**2**) is (*Z*)-14-iodotetradeca-13-en-3,5-diyn-1-ol.

### 2.2. Effects of 1 and 2 on Osteoclast Differentiation

**Figure 3:**
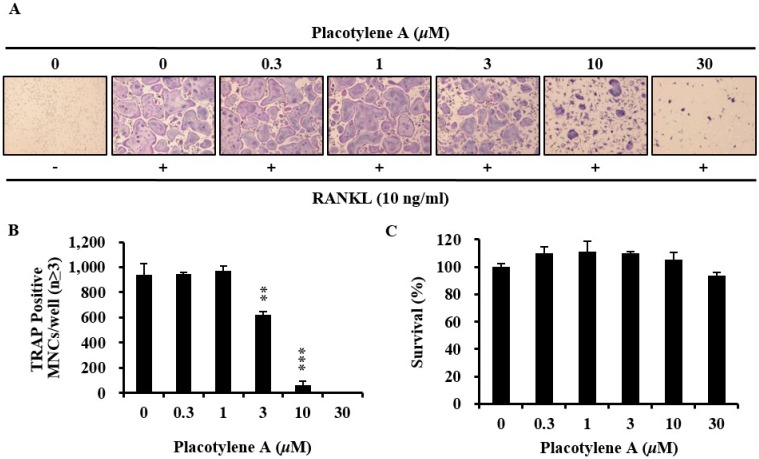
Effect of placotylene A (**1**) on receptor activator of nuclear factor-κB ligand (RANKL)-induced osteoclast differentiation. (**A**) Bone marrow-derived macrophages (BMMs) were cultured for 4 days with RANKL (10 ng/mL) and macrophage-colony stimulating factor (M-CSF) (30 ng/mL) in the presence of different concentrations of 1. The cells were fixed in 3.7% formalin, permeabilized in 0.1% Triton X-100, and stained for tartrate-resistant acid phosphatase (TRAP). (**B**) TRAP-positive cells were counted as osteoclasts (nuclei ≥3). ** P <0.01, *** P <0.001. (**C**) Cytotoxicity of 1 on BMMs. The effect of 1 on the viability on BMMs was evaluated using the CCK-8 assay.

To examine the potential role of placotylenes A (**1**) and B (**2**) during differentiation of osteoclasts, various concentrations of placotylenes A and B were added into the isolated BMMs from mouse culturing with M-CSF (30 ng/mL) and RANKL (10 ng/mL) for 4 days. **1** suppressed RANKL-induced TRAP-positive osteoclast differentiation (Figure 3A), but, **2** did not significantly suppress RANKL-induced TRAP-positive osteoclast differentiation (data not shown). Further results revealed that **1** significantly decreased the number of TRAP-positive multinucleated cells in a dose-dependent manner (Figure 3B). These results suggested that **1** could inhibit RANKL-mediated osteoclast differentiation.

**Figure 4:**
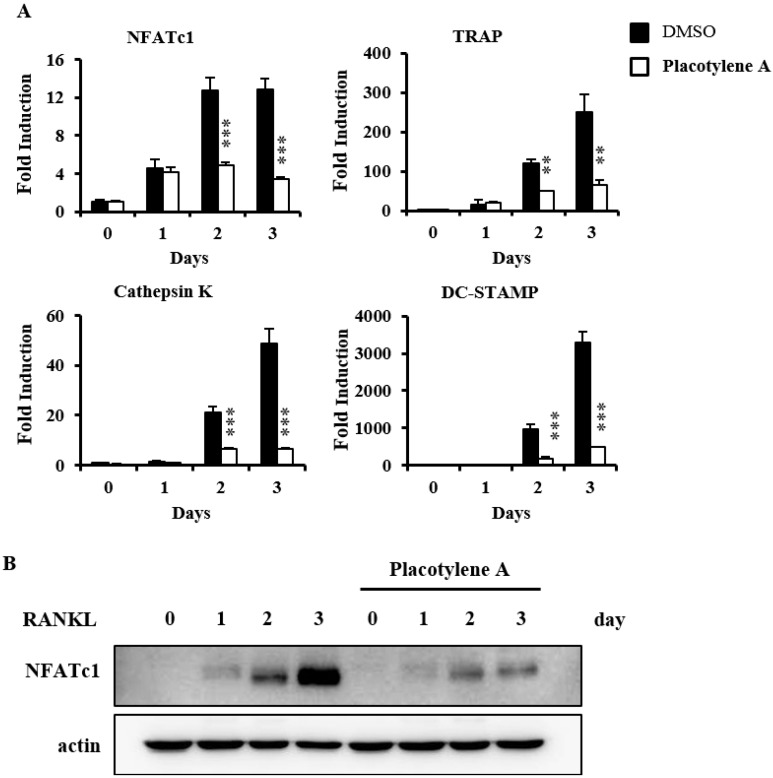
Placotylene A (**1**) abolishes RANKL-stimulated NFATc1 transcriptional and translational expression. (**A**) BMMs were pretreated with DMSO or **1** (10 μM) for 30 min and then stimulated with RANKL (10 ng/mL) for the indicated number of days. The expressed mRNA levels were analyzed by comparing real-time PCR with the DMSO control. ** P < 0.05, *** P < 0.005. (**B**) BMMs were pretreated with DMSO or **1** (10 μM) for 1 h and then stimulated with RANKL (10 ng/mL) for the indicated time. The cell lysates were analyzed by western blotting with anti-NFATc1, and anti-actin antibodies as indicated.

We confirmed the effects of **1** with the CCK-8 assay to exclude the possibility that **1** inhibits osteoclast differentiation due to its toxicity. **1** did not show any cytotoxic effects at the test doses (Figure 3C). Next, we evaluated the inhibitory effect of **1** on NFATc1 mRNA expression levels by real-time PCR. **1** significantly suppressed the mRNA expression levels of NFATc1 in BMMs treated with RANKL. **1** also dramatically suppressed the mRNA expression of osteoclast-related molecules including TRAP, DC-STAMP, and cathepsin K, during osteoclastogenesis (Figure 4A). In order to determine the translational expression level for NFATc1 in BMMs during osteoclastogenesis by RANKL, western blotting was performed in the presence of **1**. As we can see in Figure 4B, the translational expression for NFATc1 was considerably decreased.

**Figure 5:**
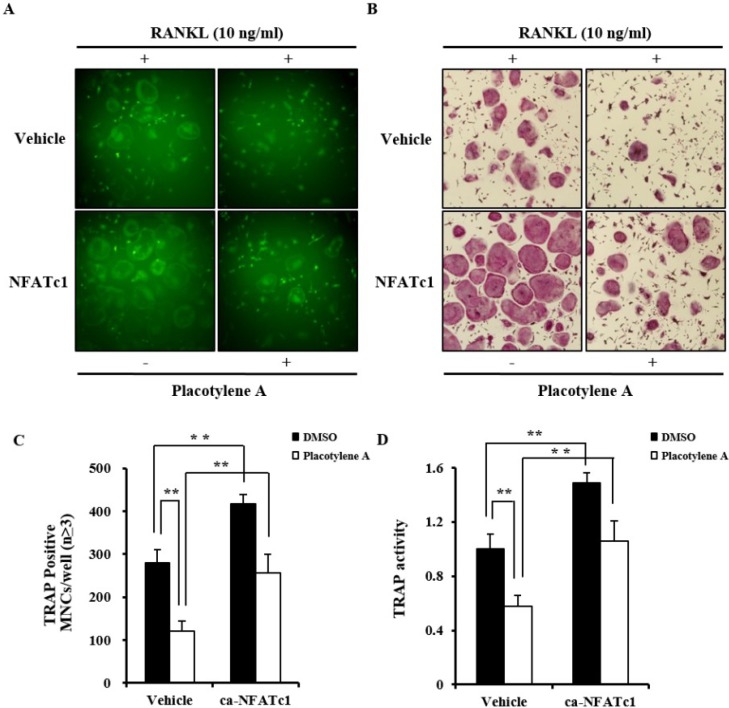
Placotylene A (**1**)-mediated inhibition of osteoclast differentiation was restored by NFATc1 over-expression. (**A**) BMMs were transduced with the indicated retroviruses harboring vehicle with GFP tag or Ca-NFATc1 expression construct with GFP tag using a retroviral method for 24 h. Infected BMMs were cultured with M-CSF (30 ng/mL) and RANKL (10 ng) for 4 days in the presence or absence of placotylene A (10 μM). The expressed GFP of the infected cells was visualized by fluorescence microscope. (**B**) TRAP-positive multinucleated osteoclasts were visualized by TRAP staining. (**C**) TRAP positive cells were counted as osteoclasts (nuclei ≥ 3). ** *P* < 0.01. (**D**) TRAP activity was measured at 405 nm. ** *P* < 0.01.

We demonstrated that the anti-osteoclastogenic action of **1** was caused by its ability to inhibit completely the transcriptional and translational expressions of NFATc1. To clarify this result, we determined whether the ectopic expression of the constitutively active form of NFATc1 (Ca-NFATc1) could rescue the **1**-mediated inhibition of TRAP-positive multinucleated osteoclasts formation. Considering GFP signaling, the infection rates of the control GFP and CA-NFATc1-GFP were similar in either BMMs treated or untreated with **1** (Figure 5A). As in the results described above, **1** noticeably inhibited the formation of TRAP-positive multinucleated osteoclasts from BMMs that express the control GFP. However, NFATc1-overexpressed BMMs were differentiated into TRAP-positive multinucleated osteoclasts even in the presence of **1** (Figure 5B). This inhibitory effect of **1** on the NFATc1-mediated formation of TRAP-positive osteoclasts was confirmed by counting the number of multinucleated osteoclasts and measuring the TRAP activity (Figure 5C,D). Taken with the results described above, it can be concluded that **1** dose-dependently inhibits osteoclast differentiation. The mechanism for the inhibitory effect of **1** involves a decrease in the transcriptional and translational expression of NFATcl. NFATc1 is the key transcriptional factor in osteoclastogenesis by RANKL and it regulates some osteoclast-specific genes [18].

## 3. Experimental Section

### 3.1. General Experimental Procedures

All NMR spectra were recorded on a Bruker Avance DPX-600 spectrometer (Bruker, Bremen, Germany) with methanol-*d*_4_ or chloroform-*d*. Mass spectral data were obtained on a JEOL, JMS-AX505WA instrument (JEOL Ltd., Tokyo, Japan). IR spectra were recorded on KBr plates by a Thermo NICOLET 570 (Thermo Fisher Scientific Inc., Waltham, MA, USA). UV spectra were also recorded in MeOH on a Scinco UVS-2100 (SCINCO, Seoul, South Korea). Solvents used in partitioning were first grade products of Dae-Jeong & Metals Co., Seoul, Korea. HPLC grade solvents from Burdick & Jackson were used for TLC and HPLC. WATERS 1525 binary HPLC pump (Milford, MA, USA) and WATERS 2489 UV/visible detector (Waters, Milford, USA) were used for purifying compounds. NMR solvents were purchased from Cambridge Isotope Laboratories (CIL) Inc. (Tewksbury, MA, USA). Recombinant mouse RANKL was purchased from R&D Systems (R&D Systems Inc., Minneapolis, MN, USA). Recombinant mouse macrophage-colony stimulating factor (M-CSF) was purchased from PEPROTECH (PeproTech Inc., Rocky Hill, NJ, USA). Cell culture medium and fetal bovine serum (FBS) were purchased from Invitrogen Life Technologies (Thermo Fisher Scientific Inc., Waltham, MA, USA). Penicillin and streptomycin were purchased Hyclone (Thermo Fisher Scientific Inc., Waltham, MA, USA). The CCK-8 assay kit obtained from Dojindo Molecular Technologies (Dojindo Molecular Technologies, Inc., Kumamoto, Japan). Anti-NFATc1 and actin antibody were obtained from SantaCruz Biotechnology (Santa Cruz Biotechnology, Inc., Dallas, TX, USA).

### 3.2. Animal Material

A species of red-orange marine sponge was collected by SCUBA near Tong-Yong City in the South Sea, Korea. The specimens were frozen at −78 °C immediately after collection. The voucher specimens were deposited in the Center for Marine Natural Products and Drug Discovery, Seoul National University, Korea. The sponge was identified as *Placospongia* species #4830 (registration no. QM G331982) by Dr. Ekins of the Queensland Museum, PO Box 3300, South Brisbane, Queensland, 4101, Australia [7].

### 3.3. Extraction, Isolation and Characterization of Placotylenes A (1) and B (2)

The frozen animal was lyophilized and then when dried (0.94 kg) was extracted three times with 50% MeOH in CH_2_Cl_2_. The dried extract (138 g) was dissolved in MeOH and washed with hexanes three times. After removal of the solvent, the MeOH-soluble fraction was resuspended in water and partitioned with EtOAc three times. The EtOAc-soluble fraction (1 g) was then fractionated into nine fractions by MPLC using a step-gradient of CH_2_Cl_2_ and Methanol as a mobile phase. The fraction three (MeOH 2% fraction) were further fractionated by RP HPLC (Phenomenex Luna C18 (2), 5 μm, 100 Å, 250 × 100 mm, 2.0 mL/min, UV = 210 nm), eluting with 40% MeCN in H_2_O to afford placotylenes A (60.5 mg) and B (5.0 mg) as colorless oils.

**Placotylene A (1):** Colorless oil; UV (MeOH) λ_max_ (log ε) 220 (3.21) nm; IR (KBr) ν_max_: 3351, 2931, 2855 cm^−1^; ^1^H, ^13^C and 2D NMR data, see Table 1; LRFABMS obsd. [M + H]^+^* m/z* 331, HRCIMS 331.0562 (calcd for C_14_H_20_OI, 331.0559).

**Placotylene B (2):** Colorless oil; UV (MeOH) λ_max_ (log ε) 217 (3.26) nm; IR (KBr) ν_max_: 3383, 2929, 2854 cm^−1^; ^1^H, ^13^C and 2D NMR data, see Table 2 LRFABMS obsd. [M + H]^+^* m/z* 331, HRFABMS 331.0561 (calcd for C_14_H_20_OI, 331.0559). (1D and 2D NMR spectra for placotylenes A and B are available in the Supplementary Information).

**Table 1:** NMR data for placotylene A (**1**, CDCl_3_) ^a^.

No.	δ_C_	δ_H_ (*J* in Hz) ^b^	COSY	HMBC
1-OH		1.96 brs		
1	60.4, CH_2_	3.75 t (6.0)	2	2, 3
2	23.6, CH_2_	2.53 t (6.0)	1	1, 3, 4, 5
3	73.8, C			
4	67.1, C			
5	65.1, C			
6	78.1, C			
7	19.0, CH_2_	2.25 t (6.0)	8	4, 5, 6, 9
8	28.5, CH_2_	1.51 m	7, 9	6, 7, 9
9	28.1, CH_2_	1.38 m	8, 10	8, 10
10	28.1, CH_2_	1.29 m	9, 11	9, 11
11	28.3, CH_2_	1.39 m	10, 12	12, 13
12	35.9, CH_2_	2.05 m	11, 13	11, 13, 14
13	146.5, CH	6.50 dt (14.3, 7.2)	12, 14	11, 12, 18
14	74.5, CH	5.98 d (14.3)	13	12, 13

^a^ Recorded at 600 MHz for ^1^H NMR and 150 MHz for ^13^C NMR. ^b^ Numbers of attached protons were determined by analysis of 2D spectroscopic data.

**Table 2:** NMR Data for placotylene B (**2**, MeOD) ^a^.

No.	δ_C_	δ_H_ (*J* in Hz) ^b^	COSY	HMBC
1	61.5, CH_2_	3.62 t (6.6)	2	2, 3
2	24.1, CH_2_	2.44 t (6.6)	1	1, 3, 4, 5
3	75.2, C			
4	67.4, C			
5	66.5, C			
6	78.3, C			
7	19.8, CH_2_	2.26 t (6.6)	8	4, 5, 6, 9
8	29.5, CH_2_	1.51 m	7, 9	6, 7, 9
9	29.7, CH_2_	1.43 m	8, 10	8, 10
10	29.7, CH_2_	1.36 m	9, 11	9, 11
11	29.0, CH_2_	1.45 m	10, 12	12, 13
12	35.7, CH_2_	2.15 m	11, 13	11, 13, 14
13	142.5, CH	6.19 dt (7.2, 7.2)	12, 14	11, 12, 18
14	82.8, CH	6.27 d (7.2)	13	12, 13

^a^ Recorded at 600 MHz for ^1^H NMR and 150 MHz for ^13^C NMR. ^b^ Numbers of attached protons were determined by analysis of 2D spectroscopic data.

### 3.4. Osteoclast Differentiation

All the experiments were performed as described in a previous paper [19]. Bone marrow cells (BMCs) were collected from 5-week-old ICR mice by flushing femurs and tibias. The BMCs collected were cultured with M-CSF (10 ng/mL) on culture dishes for 1 day, and then non-adherent BMCs were cultured with M-CSF (30 ng/mL) for 3 days. After 3 days, the adherent BMMs were isolated and treated with RANKL (10 ng/mL) and M-CSF (30 ng/mL) for 4 days. The culture media were changed every 3 days. The rate of osteoclastogenesis was analyzed by TRAP (Tartrate-resistant acid phosphatase) staining and activity assay.

### 3.5. Cytotoxicity Assay

The isolated BMMs were cultured at a density of 1 × 10^4^ cells/well on 96-well culture plates in triplicate in the presence of M-CSF (30 ng/mL) and placotylene A (at the concentrations indicated where relevant). After 3 days, the CCK-8 regent-treated cells were incubated for 4 h, and then the optical density (OD) values were measured at 450 nm.

### 3.6. Quantitative Real-Time PCR Analysis

Primers for real-time PCR analysis were designed using the online Primer3 design program [20]. Total RNAs from cultured cells were isolated with TRIzol reagent (Thermo Fisher Scientific Inc., Waltham, MA, USA) according to the manufacturer’s instructions. First-strand cDNAs were synthesized with 1 μg of total RNA, 1 μM oligo-dT18 primer, and 10 units of RNase inhibitor RNasin (Promega, Fitchburg, WI, USA) with the Omniscript RT kit (Qiagen, Venlo, Limburg, The Netherlands) following the manufacturer’s instructions. SYBR green-based qPCR was carried out with the Stratagene Mx3000P Real-Time PCR system and Brilliant SYBR Green Master Mix (Stratagene, La Jolla, CA, USA), with the first-strand cDNA and 20 pmol of primers according to the manufacturer’s protocol. PCR reaction was initiated at 95 °C for 10 min, followed by 40 cycles of 94 °C for 30 s (denaturation), 60 °C for 30 s (annealing), and 72 °C for 30 s (extension). This was followed by the generation of PCR-product temperature-dissociation curves at 95 °C for 1 min, 55 °C for 30 s, and 95 °C for 30 s. All PCR reactions were run in triplicate, and then data were analyzed by 2^−ΔΔCT^ method [21]. Glyceraldehyde-3-phosphate dehydrogenase (GAPDH) was used as an internal standard. 

### 3.7. Western Blotting Analysis

Cultured cells were washed with ice-cold phosphate-buffered saline (PBS) and lysed in lysis buffer (50 mM Tris-HCl, 150 mM NaCl, 5 mM ethylenediaminetetraacetic acid (EDTA), 1% Triton X-100, 1 mM sodium fluoride, 1 mM sodium vanadate, and 1% deoxycholate) supplemented with protease inhibitors. Cell lysates were boiled in sodium dodecyl sulfate (SDS) sample buffer for 5 min and loaded on 10% or 12% SDS-polyacrylamide gel electrophoresis (PAGE) gels. After proteins were separated in the gels, they were transferred on a polyvinylidene difluoride membrane (Millipore Corporation, Billerica, MA, USA). The membrane was washed with TBST (10 mM Tris-HCl pH 7.5, 150 mM NaCl, and 0.1% Tween 20) and incubated in the blocking solution (TBST with 5% skim milk). The membrane was probed with the indicated primary antibody, washed for 30 min three times, and then incubated with secondary antibody conjugated to horseradish peroxidase for 2 h, and washed for 30 min three times. The membrane was developed with Super-Signal West Femto Maximum Sensitivity Substrate (Thermo Fisher Scientific Inc., Waltham, MA, USA) using the LAS-3000 luminescent image analyzer (Fuji Photo Film Co. Ltd., Tokyo, Japan).

### 3.8. Forced Expression of NFATc1 Using Retroviral Vectors

Retrovirus packaging was carried out as the previous report [22]. In brief, to isolate retrovirus, pMX-IRES-GFP (retrovirus vector, GFP; green fluorescent protein) and pMX containing constitutively active (CA)-NFATc1 were transiently transfected into Plat-E cells (platinum-E retrovirus packaging cell line; Cell Biolabs, Inc., San Diego, CA, USA) with Lipofectamine 2000 (Thermo Fisher Scientific Inc., Waltham, MA, USA) according to the manufacturer’s protocol. After transfection, viral supernatant was collected from the culture medium after 48 h. BMMs were incubated with viral supernatant for 8 h in the presence of polybrene (10 μg/mL). The infection efficiency of the retrovirus was determined by GFP expression. After infection, BMMs were induced to differentiate in the presence of M-CSF (30 ng/mL) and RANKL (5 ng/mL) for 4 days.

### 3.9. Statistical Analysis

Differences in data between groups are presented as the mean ± S.D. of 3 replicates. Statistical differences were analyzed using Student’s *t*-test. Probability values less than 0.05 were considered significant (*P* values * <0.05, ** <0.01, *** <0.001).

## 4. Conclusions

We have isolated very rare iodinated polyacetylenes, placotylene A (**1**) and B (**2**), from a marine sponge *Placospongia* sp. **2** could be an artifact of **1** as a result of *cis*-*trans* isomerization by light or temperature during the isolation process. We have previously reported two phosphoiodyns with (*E*)-14-iodotetradeca-13-en-3,5-diyn-1-yl moiety from the same sponge; however, the regiomer of this moiety was not observed in the molecules. **1** has an inhibitory effect on osteoclast differentiation. **1** may inhibit the differentiation of osteoclast by decreasing the transcriptional and translational expression of NFATc1, which is a critical factor in the regulation of osteoclastogenesis. This could provide a basis of drug discovery for osteoporosis involving a new structural scaffold of osteoclast differentiation inhibitors.

## Supplementary Files

Supplementary File 1 Supplementary Information (PDF, 342 KB)
