# Supplementary material for: Placotylene A, an Inhibitor of the Receptor Activator of Nuclear Factor-κB Ligand-Induced Osteoclast Differentiation, from a Korean Sponge Placospongia sp
Source: Mar Drugs. 2014 Apr 3;12(4):2054–65. doi: 10.3390/md12042054 (PMC4012465; doi:10.3390/md12042054)
Supplement: Supplementary File 1 — Supplementary Information (PDF, 342 KB) [file marinedrugs-12-02054-s001.pdf]

Supplementary Information

Figure S1. ^{1}H NMR spectrum (600 MHz) of placotylene A (**1**) in chloroform-*d*.
Figure S2. ^{13}C NMR spectrum (150 MHz) of placotylene A (**1**) in chloroform-*d*.
Figure S3. COSY spectrum (600 MHz) of placotylene A (**1**) in chloroform-*d*.
Figure S4. HSQC spectrum (600 MHz) of placotylene A (**1**) in chloroform-*d*.
Figure S5. HMBC spectrum (600 MHz) of placotylene A (**1**) in chloroform-*d*.
Figure S6. ^{1}H NMR spectrum (600 MHz) of placotylene B (**2**) in MeOD.
Figure S7. ^{13}C NMR spectrum (150 MHz) of placotylene B (**2**) in MeOD.
Figure S8. COSY spectrum (600 MHz) of placotylene B (**2**) in MeOD.
Figure S9. HSQC spectrum (600 MHz) of placotylene B (**2**) in MeOD.
Figure S10. HMBC spectrum (600 MHz) of placotylene B (**2**) in MeOD.

Figure S1. ^{1}H NMR spectrum (600 MHz) of placotylene A (**1**) in chloroform-*d*.

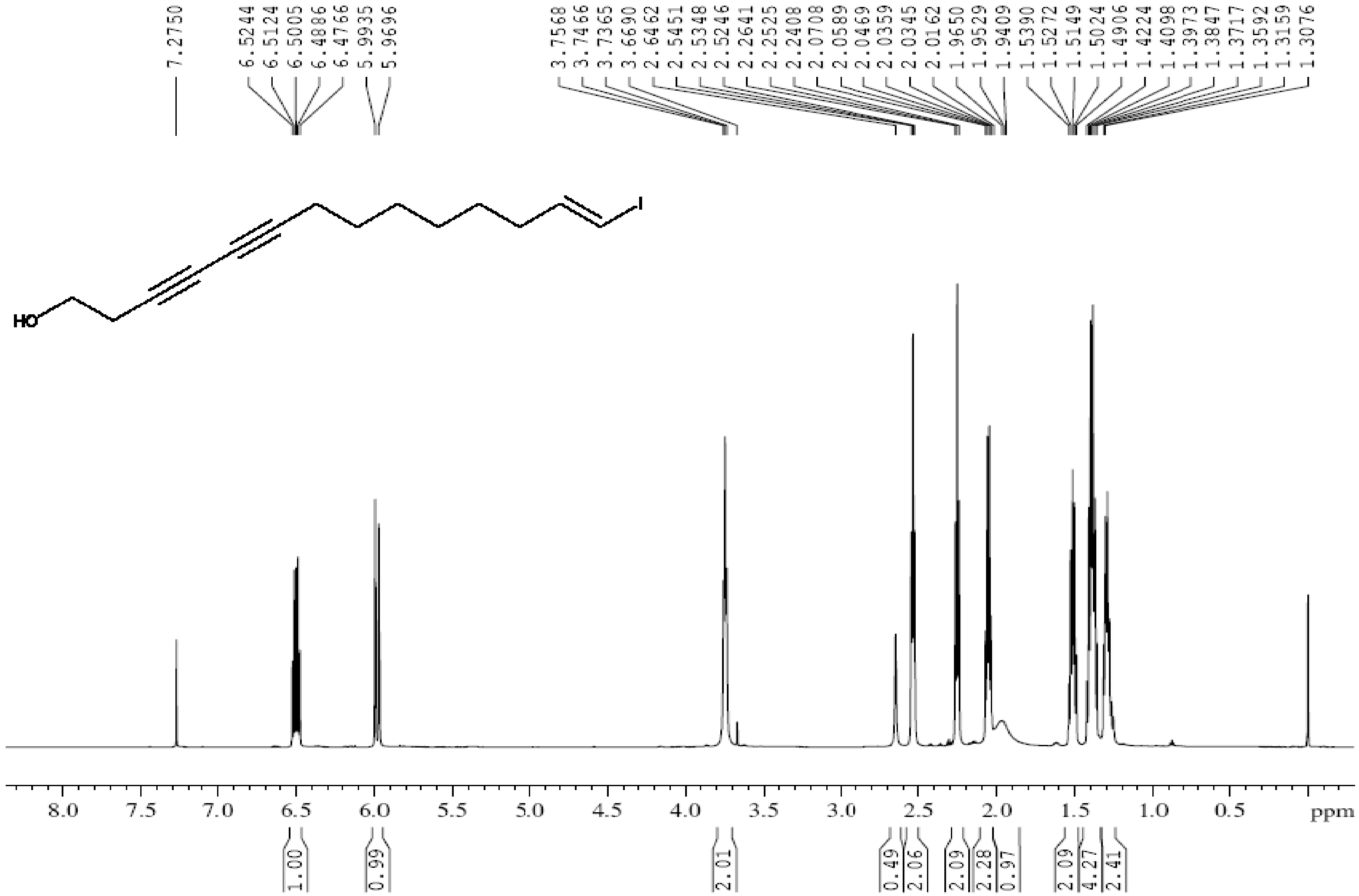

Figure S2. ^{13}C NMR spectrum (150 MHz) of placotylene A (**1**) in chloroform-*d*.

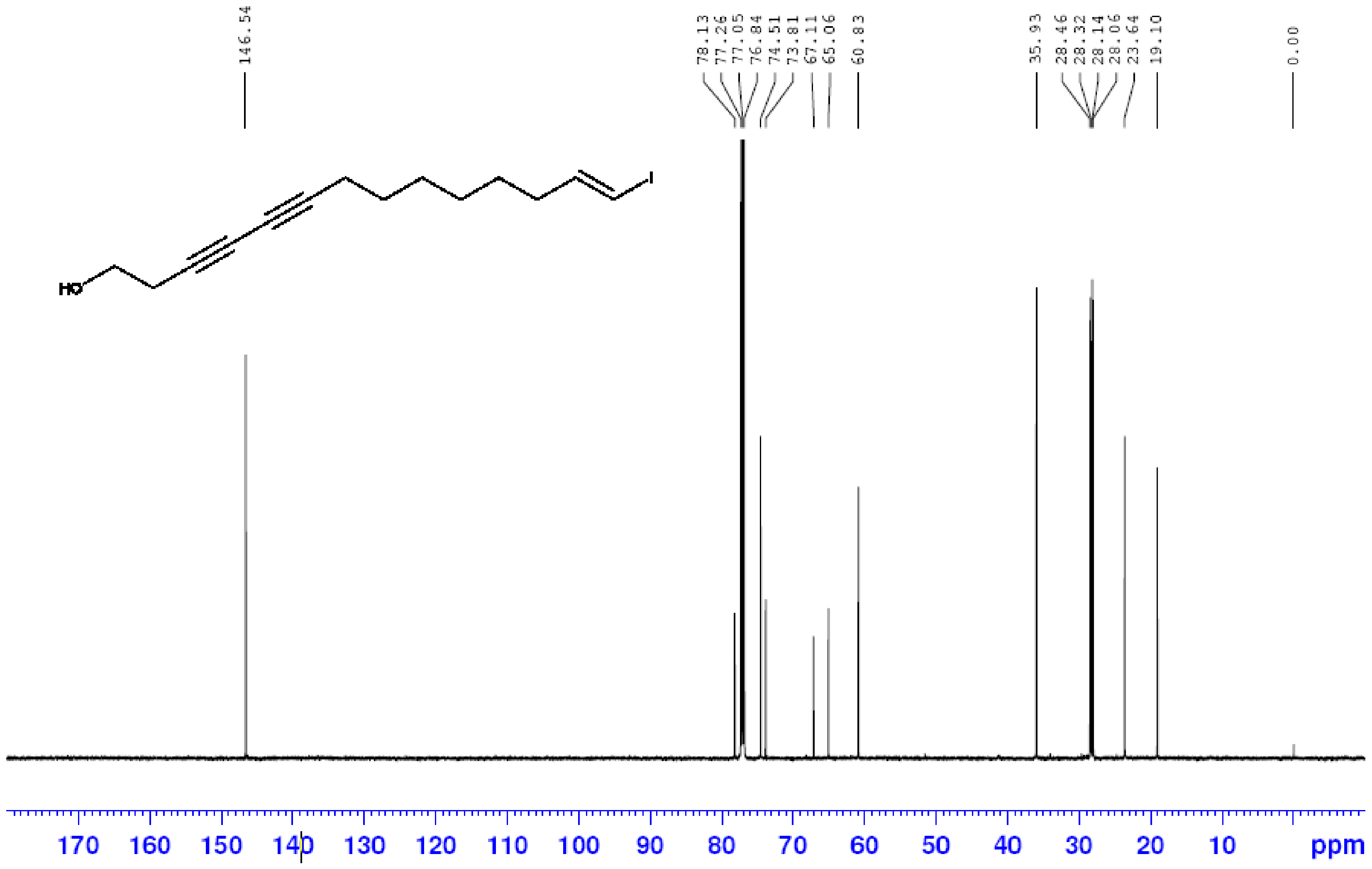

Figure S3. COSY spectrum (600 MHz) of placotylene A (**1**) in chloroform-*d*.

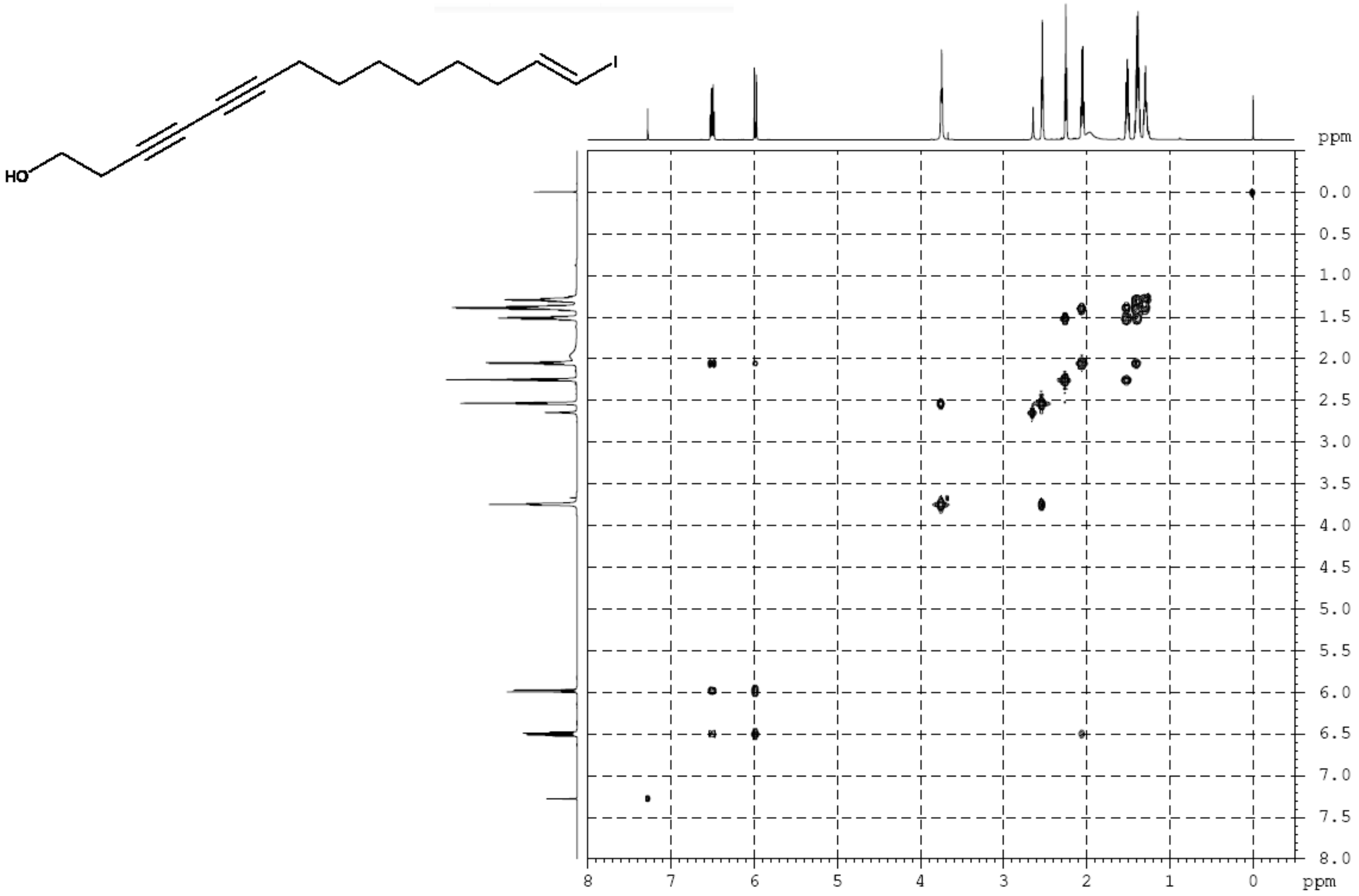

Figure S4. HSQC spectrum (600 MHz) of placotylene A (**1**) in chloroform-*d*.

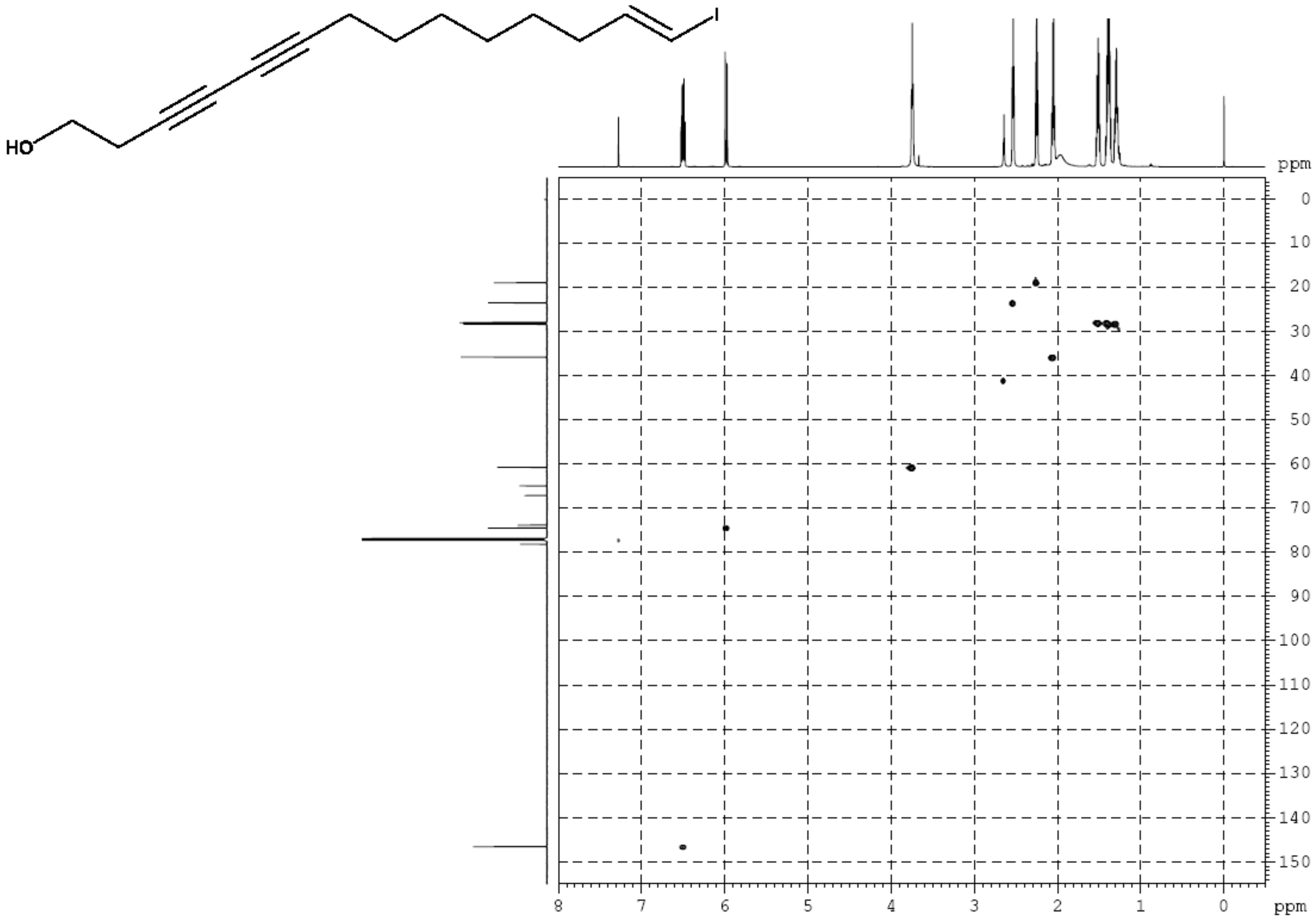

Figure S5. HMBC spectrum (600 MHz) of placotylene A (**1**) in chloroform-*d*.

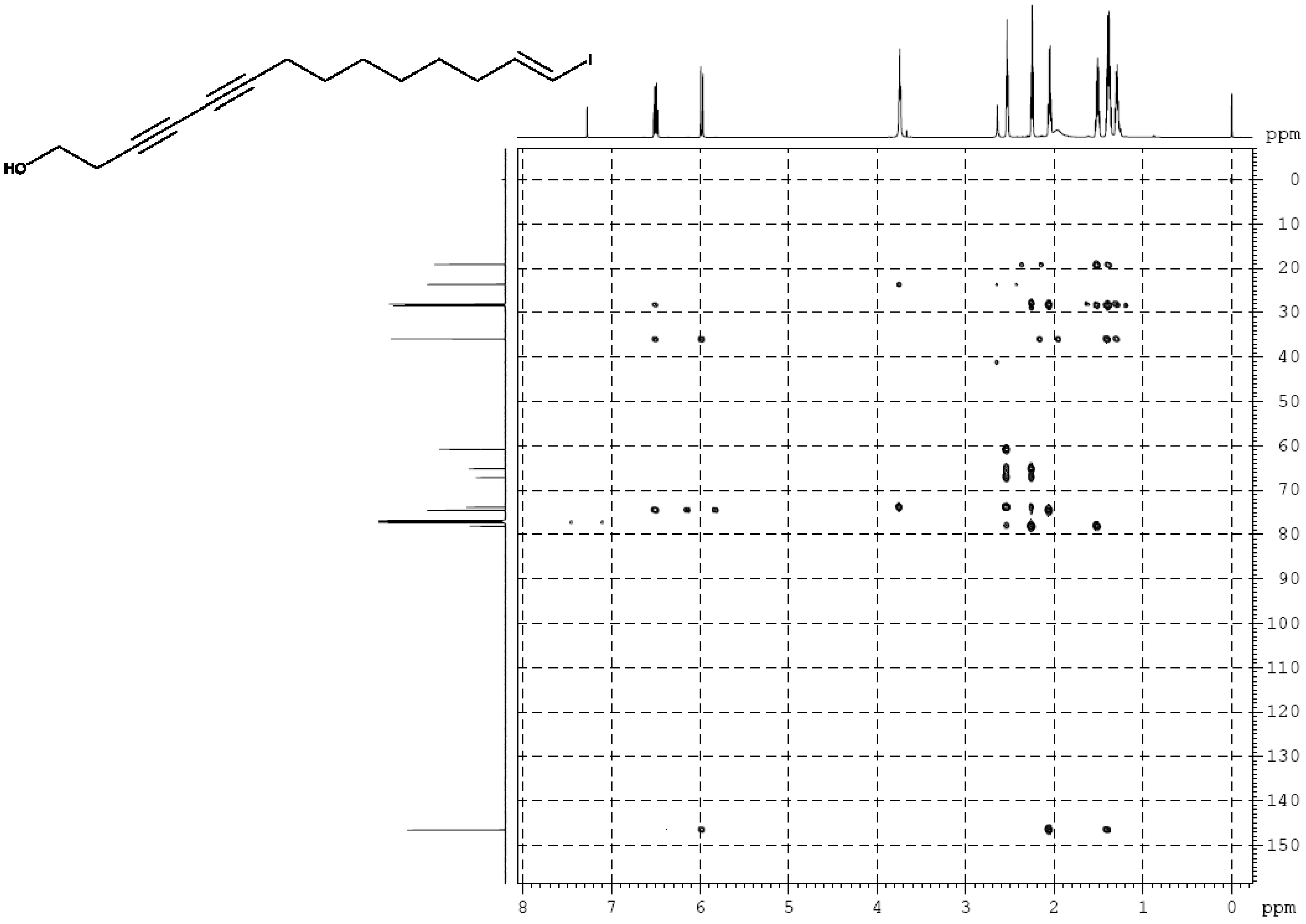

Figure S6. ^{1}H NMR spectrum (600 MHz) of placotylene B (**2**) in MeOD.

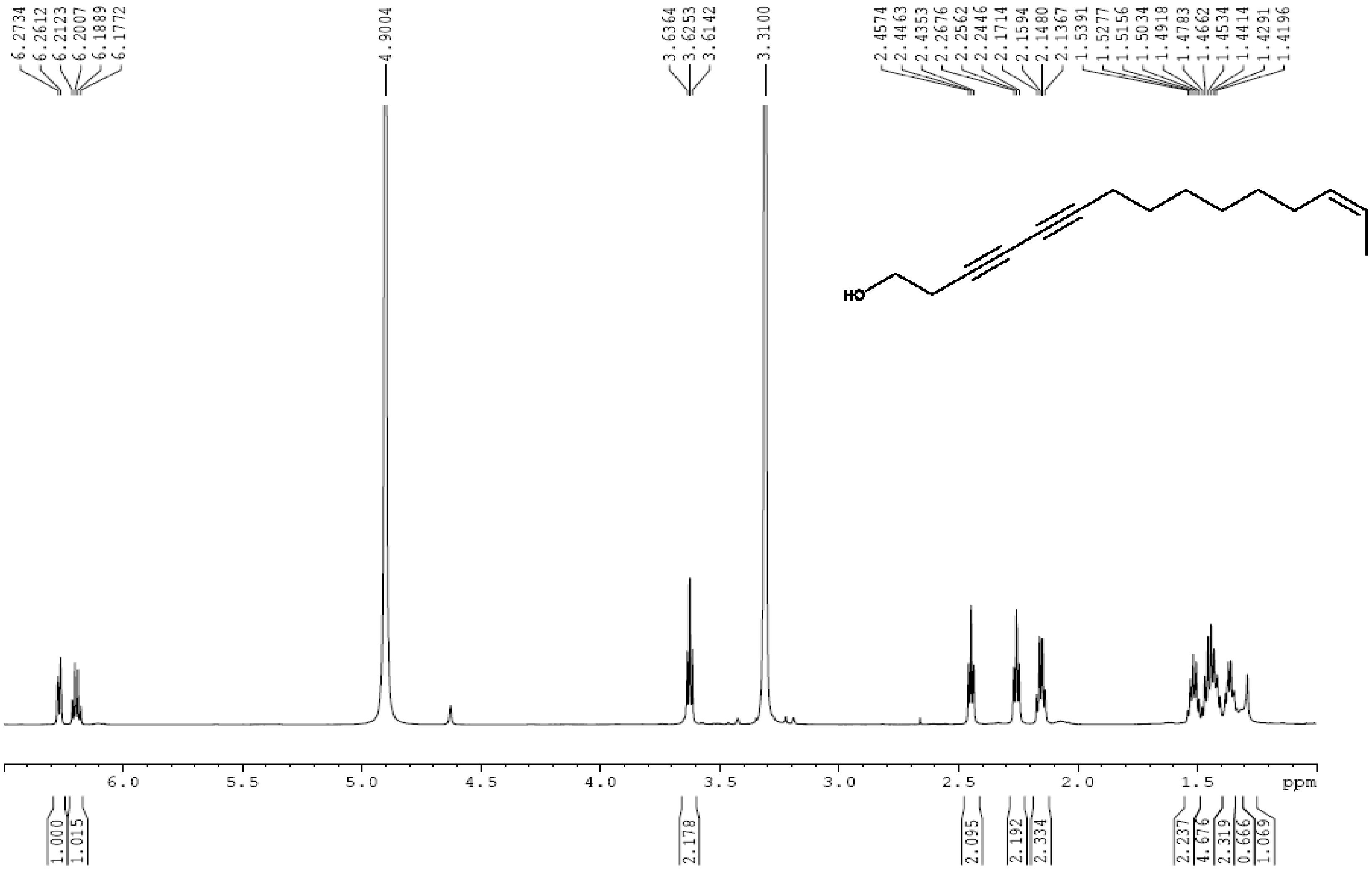

Figure S7. ^{13}C NMR spectrum (150 MHz) of placotylene B (**2**) in MeOD.

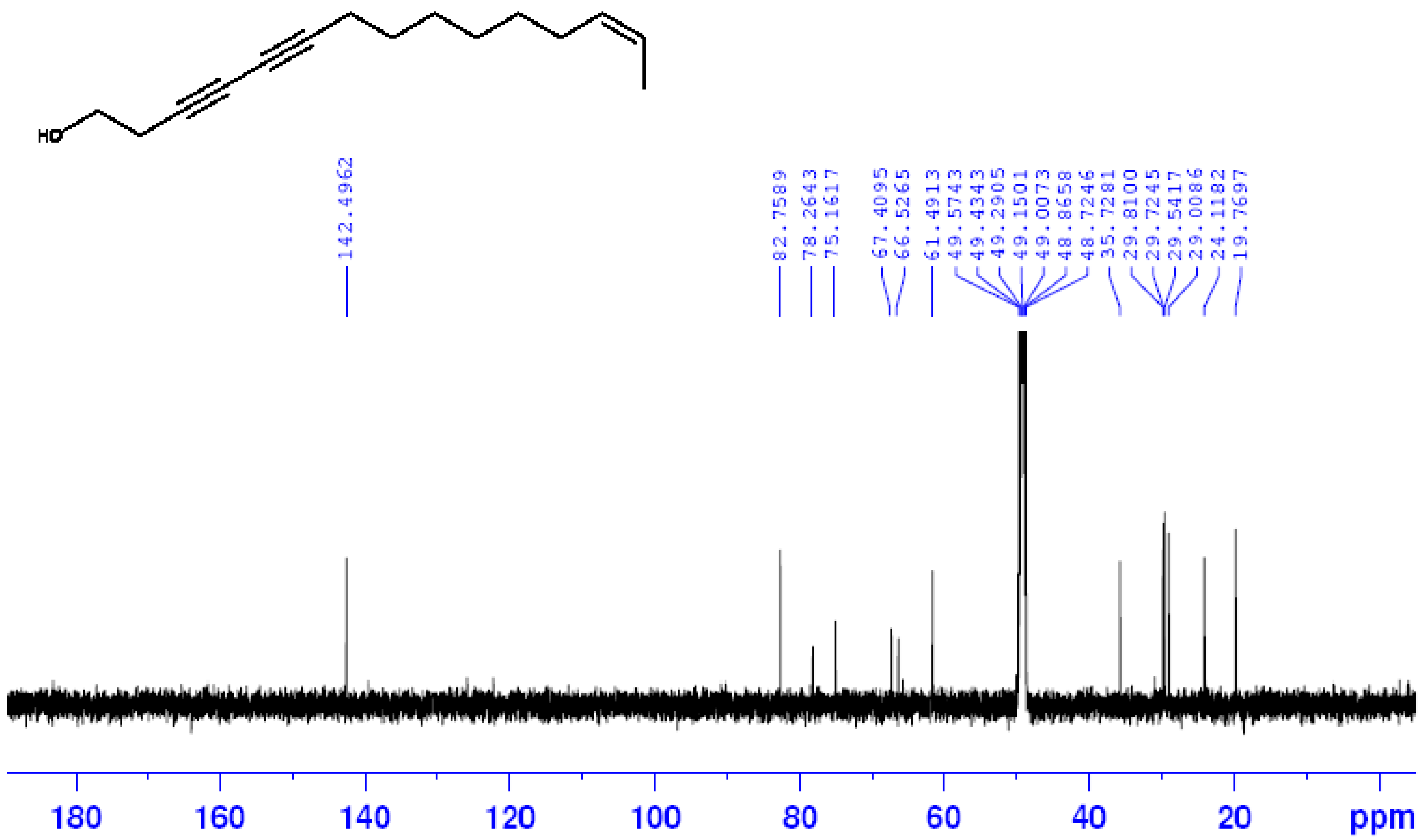

Figure S8. COSY spectrum (600 MHz) of placotylene B (**2**) in MeOD.

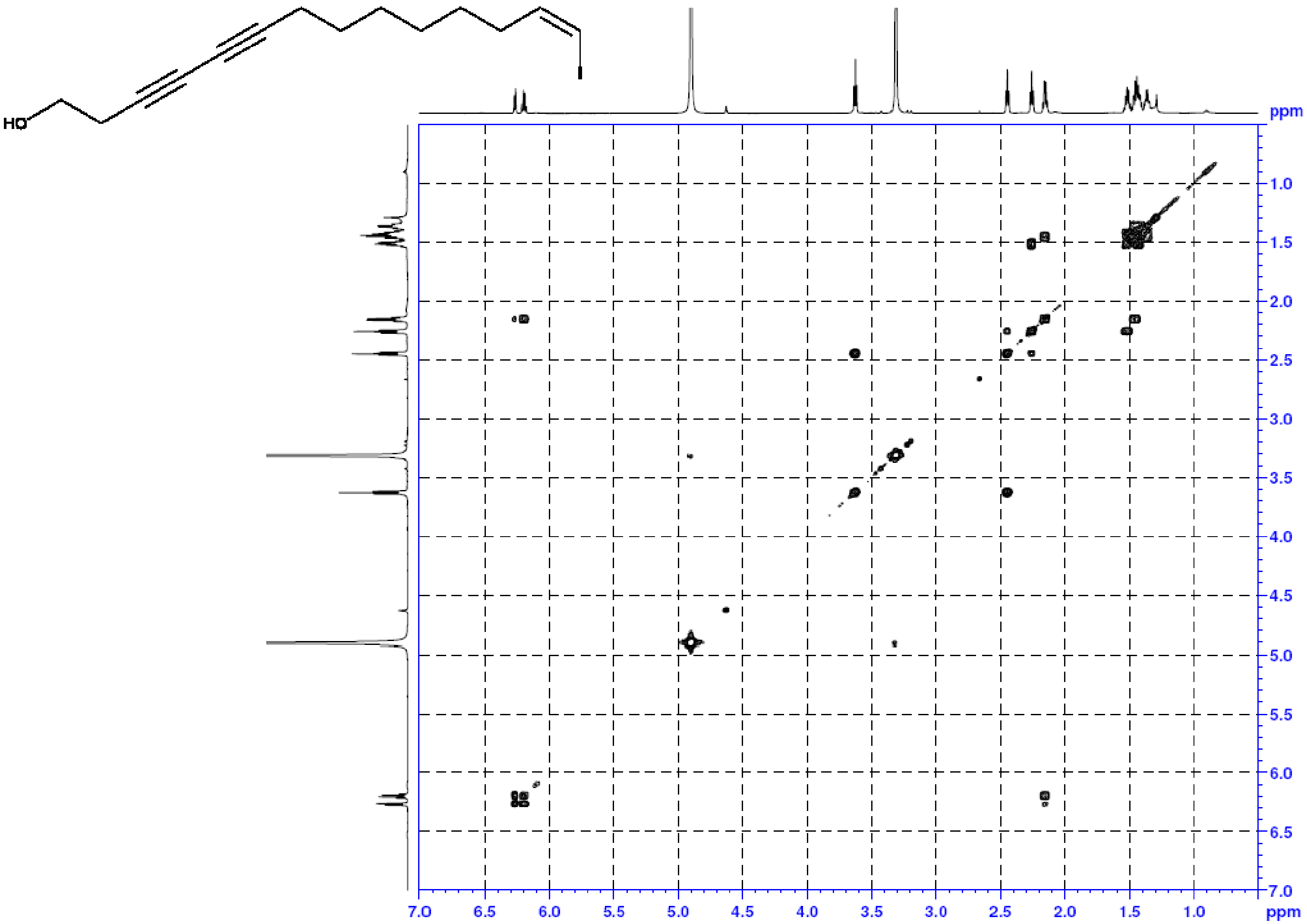

Figure S9. HSQC spectrum (600 MHz) of placotylene B (**2**) in MeOD.

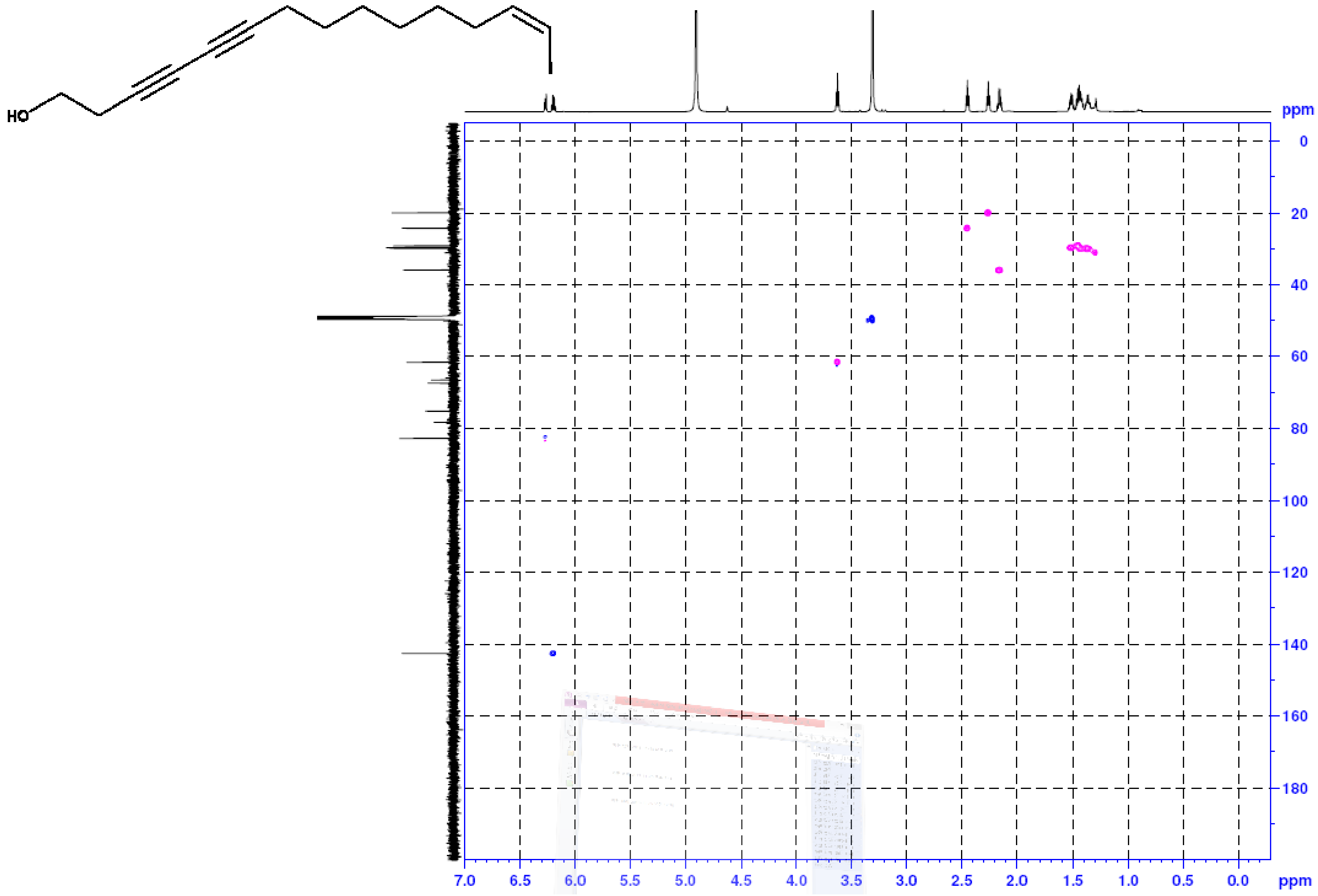

Figure S10. HMBC spectrum (600 MHz) of placotylene B (**2**) in MeOD.

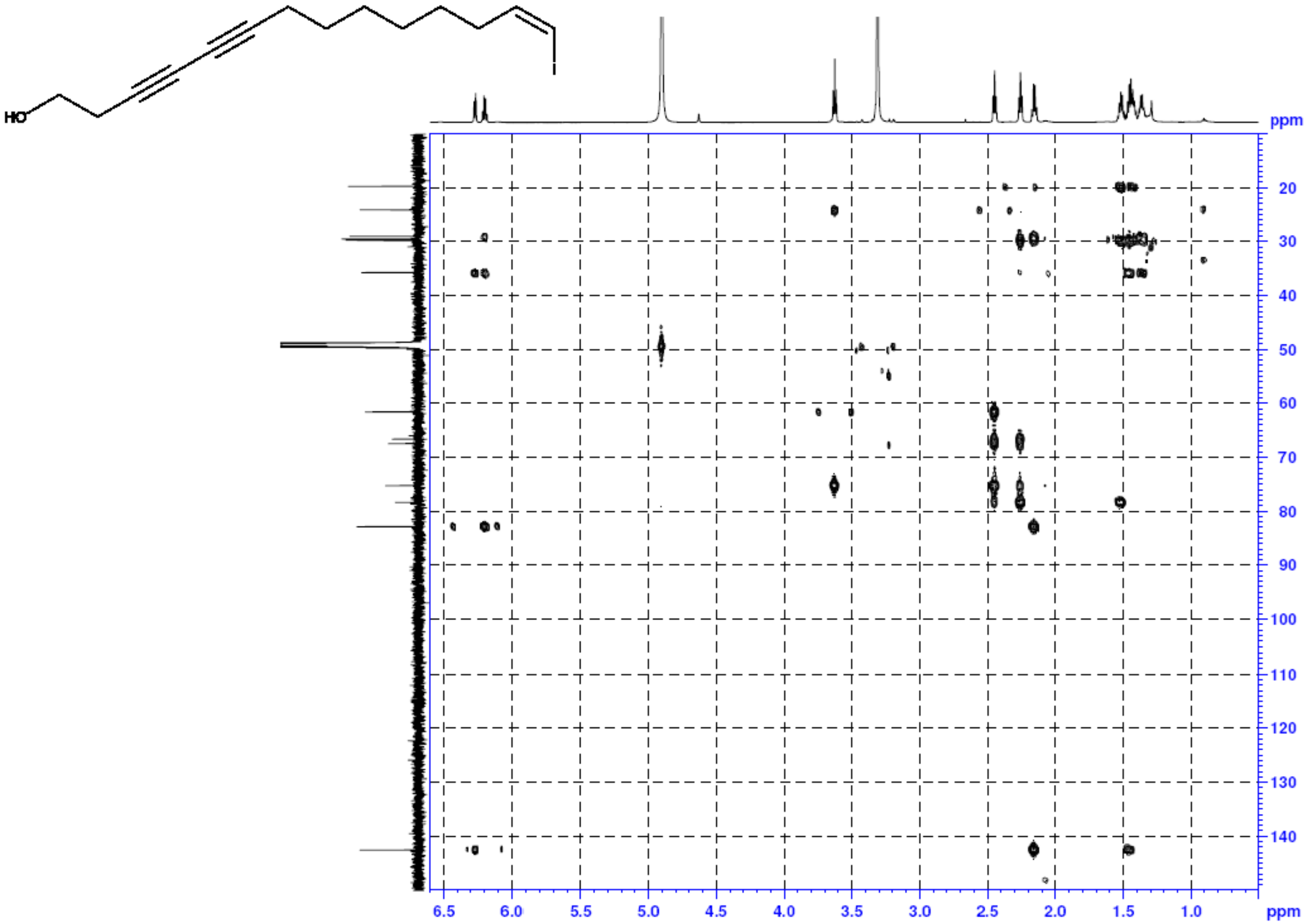

© 2014 by the authors; licensee MDPI, Basel, Switzerland. This article is an open access article distributed under the terms and conditions of the Creative Commons Attribution license (http://creativecommons.org/licenses/by/3.0/).
